# The Mechanical Glass Transition Temperature Affords a Fundamental Quality Control in Condensed Gels for Innovative Application in Functional Foods and Nutraceuticals

**DOI:** 10.3390/foods14122098

**Published:** 2025-06-14

**Authors:** Vilia Darma Paramita, Naksit Panyoyai, Stefan Kasapis

**Affiliations:** 1 Department of Chemical Engineering, State Polytechnic of Ujung Pandang, Tamalanrea, Makassar 90245, Indonesia; viliadarma@poliupg.ac.id; 2 Department of Agroindustry, Rajabhat Chiang Mai University, Chiang Mai 50330, Thailand; naksit_pan@cmru.ac.th; 3 School of Science, RMIT University, Bundoora West Campus, Plenty Road, Melbourne, VIC 3083, Australia

**Keywords:** mechanical glass transition temperature, condensed hydrocolloid gels, functional foods, nutraceuticals, delivery vehicles, encapsulating agents

## Abstract

A subject of increasing fundamental and technological interest is the techno- and bio-functionality of functional foods and nutraceuticals in high-solid gels. This encompasses the diffusion of natural bioactive compounds, prevention of oxidation of essential fatty acids, minimization of food browning, and the prevention of malodorous flavour formation in enzymatic and non-enzymatic reactions, to mention but a few. Textural and sensory considerations require that these delivery/encapsulating/entrapping vehicles are made with natural hydrocolloids and co-solutes in a largely amorphous state. It is now understood that the mechanical glass transition temperature is a critical consideration in monitoring the performance of condensed polymer networks that incorporate small bioactive compounds. This review indicates that the metastable properties of the rubber-to-glass transition in condensed gels (as opposed to the thermodynamic equilibrium in crystalline lattices) are a critical parameter in providing a fundamental quality control of end products. It appears that the “sophisticated synthetic polymer research” can provide a guide in the design of advanced biomaterials for targeted release or the prevention of undesirable byproducts. Such knowledge can assist in designing and optimizing functional foods and nutraceuticals, particularly those including vitamins, antioxidants, essential fatty acids, stimulants for performance enhancement, and antimicrobials.

## 1. Introduction

A gel is a structure composed of cross-linked biopolymer molecules that form a tangled, interconnected network supporting a liquid medium. The gelation forms a continuous network with the polymer chains, providing strength and solid-like properties throughout the material [1]. The strength of gels is mostly stabilized by physical interactions such as hydrogen bonds, electrostatic forces, and hydrophobic associations [2]. The strong physical hydrogels, like those with lamellar microcrystals, glassy nodules, or double and triple helices that may or may not further aggregate, exhibit durable bonds between polymer chains that remain stable under certain thermal, shear, and solvent/plasticization conditions. On the other hand, weak physical hydrogels have temporary connections formed by relatively unstable hydrogen bonds and ionic associations, experiencing continuous disruption and deformation under environmental conditions [3]. The weak gels are typically thermoreversible, but still capable of incorporating significant amounts of water (hydrogels), air (aerogels), or oil (oleogels) within their three-dimensional structure [4].

Food gels can also be classified into tough/high-solid gels, smart/stimuli-responsive gels, and aerogels based on factors such as water content, thermal characteristics, and interactions [4]. Tough gels display attributes like double/composite networks, organic–inorganic hybrids, and both chemical and physical crosslinks, featuring transformable domains and crystalline structures [5]. Smart/stimuli-responsive gels, on the other hand, are sensitive to aspects including pH, ionic strength, temperature, light, enzymes, electric/magnetic fields, and biological circumstances such as CO_2_, glucose, and more [6]. Meanwhile, aerogels possess the characteristics of a high specific surface area, high degree of porosity, ultralow thermal conductivity, and low density [7].

Other useful characteristics of food gels include viscoelastic properties, combining both viscous and elastic contributions, which allows them to deform under stress or heat, but eventually return to their original shape at thermodynamic equilibrium [1]. This behaviour makes them valuable in various applications across industries, including food, pharmaceuticals, and cosmetics [8]. In the food sector, gels enhance texture, stability, and mouthfeel, significantly influencing consumer satisfaction and product quality. Their ability to adapt to different environmental conditions and incorporate various substances further enhances their versatility in both culinary and nutraceutical applications [9,10]. Modern food design utilizes gels in various techno- and bio-functional ways, including creating the desired sensory texture of food, enhancing the stability of foams and emulsions for longer shelf life, aiding in the dispersion and suspension of bioactive particles, preserving moisture, and reducing water syneresis [4,10,11]. Gels help in trapping and releasing flavours, replacing fats and sugar, adding dietary fibre, and increasing food volume to promote satiety in the digestive system [12]. More recently, gels have also aided in forming intricate shapes in foods through 3D and 4D printing technologies [13].

In particular, there is increasing research in condensed soft matter, which is currently considered a cutting-edge evolving field. The gel structure of such systems is designed to contain more than 70% *w*/*w* solid content, compared to common hydrogels of 20% *w*/*w* solid or less [14,15]. The main components of high-solid gels are natural hydrocolloids such as proteins and polysaccharides, as well as co-solutes like ionic counterions, glucose syrup, sucrose, polydextrose, sorbitol, and water [16,17]. This high-solid gel system exhibits liquid-like flow and solid-like behaviour depending on changes in temperature or pressure [18,19]. Previous research on high-solid food gels has shown that condensed starch gels inhibit the activity of the enzyme a-amylase in the digestion of amylose molecules [20], while condensed protein gels inhibit the growth of food pathogens [21]. The transition of the high-solid gel from a liquid-like state to a solid-like state was also shown to regulate the release of bioactive compounds, such as caffeine, vitamins, and essential fatty acids in a model food system [22,23,24,25]. Recent work by Ikasari et al. (2025) reported that the oxidation reaction of polyunsaturated fatty acids, which is a chain reaction leading to rancidity and the loss of nutritional value in food, can be suppressed by the glassy state in high-solid polysaccharide systems [26].

Therefore, this article provides an overview of food gels, highlighting their key characteristics and the mechanisms involved in their formation. It specifically examines the physical properties of high-solid gels during the glass transition process and their impact on food structure. Additionally, the article explores the mechanical properties of gel systems with limited water content at various temperatures and their role in enhancing the techno- and bio-functionality of food systems. The review also focuses on the controlled release of bioactive compounds and the retardation of chemical reactions, including lipid oxidation and the Maillard reaction, in high-solid systems. It explores their potential applications in food preservation, functional foods, and nutraceuticals while also comparing these uses to current trends in the application of gels as delivery vehicles in the biomedical and pharmaceutical fields.

## 2. Overview of Low- and High-Solid Gels

High-solid gels are primarily structured through the network formation of gelling agents widely utilized in the food industry [27]. These agents are naturally occurring hydrocolloids, including whey protein [28], gelatin [29], pectin [30], carrageenan [31], agarose [32], alginate [33], and chitosan [34], to mention but a few. They exhibit essential properties such as edibility, biocompatibility, biodegradability, and nutritional functionality, making them valuable components in food formulation for end product development [35]. The gelation process occurs following dispersion in water, with the temperature and water content regulating the properties of the system. Water acts as a medium that facilitates the dissolution of hydrocolloids and their water absorption during heating, leading to the transition from solution to gel. This transformation typically promotes intermolecular interaction, leading to aggregation and the formation of a three-dimensional structure that supports a certain functionality [36]. Overall, the mechanisms of gel formation have been extensively discussed in the literature, highlighting various factors and molecular processes that contribute to these thermodynamic transitions (Table 1) [4,6,10,36,37].

**Figure 1:**
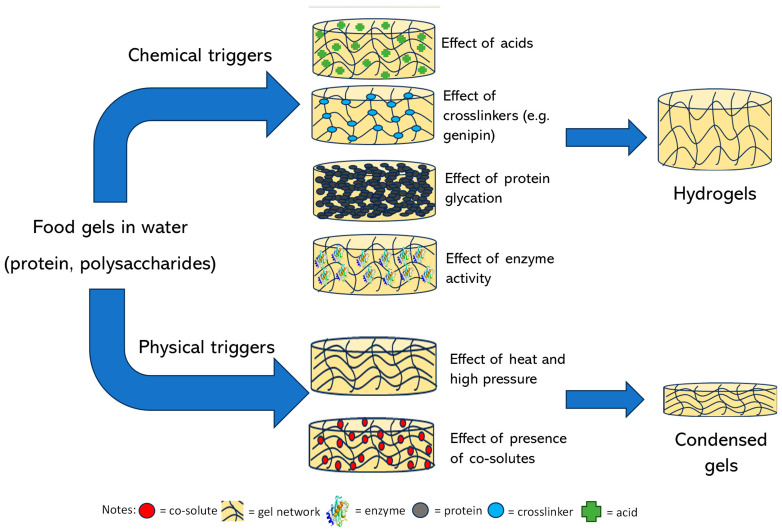
Formation mechanism of food gels.

Gelation reactions for high-solid systems can generally be divided into two main types: those triggered by physical forces such as heat and pressure, and those initiated by chemical mechanisms, including acidic and enzymatic processes, protein glycation, or the Maillard reaction, etc. Physical crosslinking, induced by external stimuli, leads to the formation of non-covalent forces between intermolecular or intramolecular chain segments, such as hydrogen bonding, ionic interactions, hydrophobic clusters, van der Waals forces, or intermolecular entanglement (Figure 1) [35].

In physically induced high-solid gels, the water content is limited to no more than 30% by incorporating simple and complex carbohydrates, such as glucose syrup, sucrose, and polydextrose. This formulation strengthens the gel structure while maintaining its thermally reversible properties after heating and cooling [22]. Conversely, the chemical crosslinking mechanism involves adding a crosslinker to the solution of the gelling agent before drying the mixture. This crosslinking allows the gel sheet to retain its functionality as a high-solid medium [38]. Due to the condensed structure of the high-solid gels, they are, therefore, suitable for encapsulating/entrapping biologically and technologically important components, including vitamins, essential fatty acids, and amino acids in the glassy or rubbery state. Such incorporation of thermally labile substances in high-solid matrices prevents nutrient degradation.

In the case of deacylated gellan gum, two distinct types of networks are observed that can be classified into enthalpic or entropic structures. The enthalpic network relies on energy-driven intermolecular interactions, also known as polymeric junction zones, exhibiting the viscoelasticity of a hydrogel, whereas an entropic network is stabilized primarily by the configurational entropy of flexible chains between junction zones and topological constraints, exhibiting rubber-like elasticity that undergoes vitrification during cooling or increasing the frequency of oscillation [39]. At low levels of co-solute, gellan tends to form strong, enthalpically driven gels via hydrogen bonding and ionic crosslinking (especially with divalent cations like calcium), leading to the formation of ordered double helices between the polysaccharide chains. The outcome is strong, well-structured systems that exhibit thermal stability and brittleness [40,41,42].

**Table 1:** Food gels and their mechanism of formation.

Source	Chemical Binding Blocks	Mechanism of Gel Formation	References
Plant- and algae-based gels			
Agar	The agar is composed of α (1–4)-3, 6-anhydro-L-galactose, and β 9(1–3)-D-galactose units.	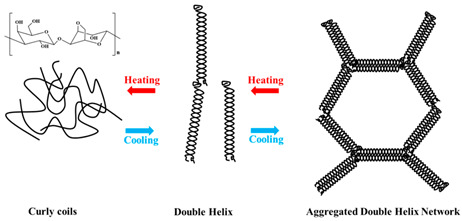 E.g., effect of heating and cooling during gel helix formation.	[43] and reprinted from [32] with permission from MDPI
Alginate (alginic acid)	Linear copolymer with blocks of (1–4)-linked *β*-D-mannuronate and its C-5 epimer *α*-L-guluronate residues, covalently linked together in different sequences.	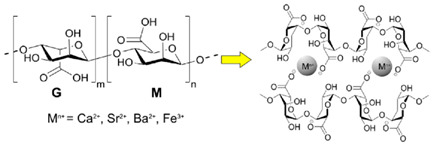 E.g., alginic acid and the ionic crosslinking of alginate via multivalent metal cations (M^n+^), involving α-L-guluronic acid (G) and β-D-mannuronic acid (M) units to form “egg box” gels.	Reprinted from [33] with permission from MDPI
κ-, ι-, and λ- carrageenan	Sulphated D-galactose and L-anhydrogalactose.	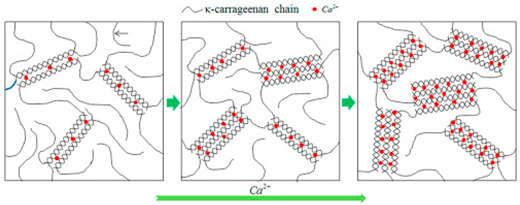 E.g., Ca^2+^ induced gelation in k-carrageenan to form double helices.	Reprinted from [31] with permission from Elsevier
Cellulose (carboxymethyl-cellulose)	Homo-polymer of *β* (1, 4) D-glucose.	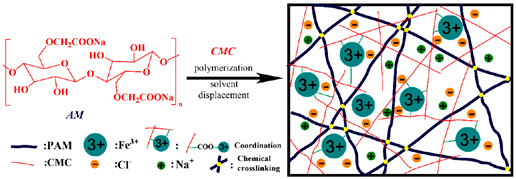	Reprinted from [44] with permission from MDPI
Corn protein (Zein)	A major corn-derived prolamin consists mainly of α, β, γ, and δ forms, with α-zein being the most common. Its composition includes both polar (e.g., glutamic acid, tyrosine) and non-polar (e.g., proline, leucine) amino acids.	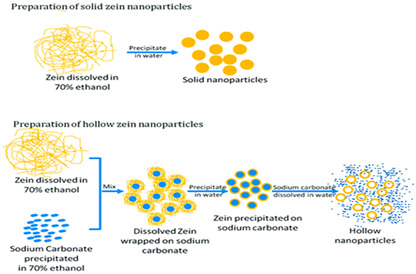 E.g., effect of solvent and sodium carbonate in zain nanoparticle gels.	[45] and reprinted from [46] with permission from BMC
Guar gum	Linear chain of galactomannan unit.	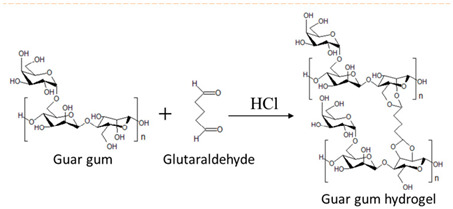 E.g., guar gum gel formation in the presence of glutaraldehyde.	Reprinted from [47] with permission from Elsevier
Konjac mannan	Glucomannan is a heteropolysaccharide consisting of D-glucose (G) and D-mannose (M), linked by *β* -D-1,4 bonds with a G/M ratio of 1 to 1.6.	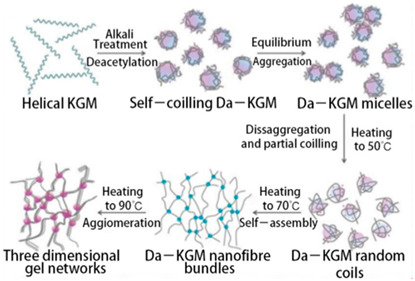 E.g., alkali–induced gelation mechanism of konjac mannan (KGM).	Reprinted from [48] with permission from MDPI
Mung bean protein	Globulins (60%, vicilin-type 8S with MW 26–60 kDa); albumins (25%, MW 24 kDa); other globulins including basic-type 7S and legumin-type11S.	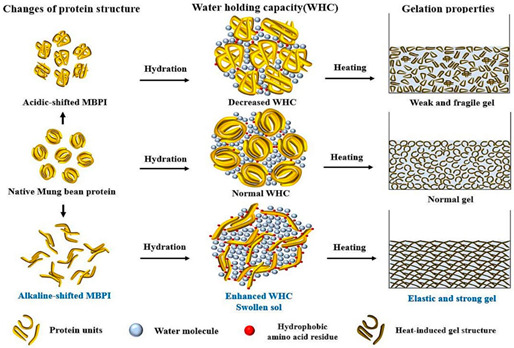	Reprinted from [49] with permission from Elsevier
Pea protein	The protein is primarily composed of globulins, with legumin (11S, a hexamer with a molecular weight of 320–380 kDa) and vicilin (7S, a trimer with a molecular weight of 150–170 kDa, which lacks cysteine residues) being the dominant types. A smaller proportion consists of convicilin (molecular weight 290 kDa).	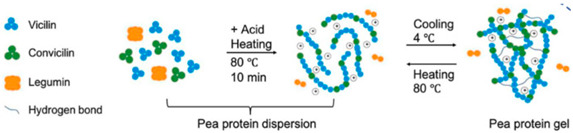 E.g., effect of acid and heating and cooling processes during gel helix formation.	Reprinted from [2] with permission from MDPI
Pectin (high methoxyl, HM and low methoxyl, LM)	Linear polymer of partly esterified *α*-(1–4)-linked D-galacturonic and mannuronic acid. Sometimes galacturonic acid is replaced by rhamnose, galactose, and arabinose.	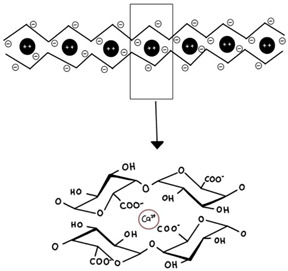 E.g., the low-methoxy pectin “egg-box” model illustrates the gelling mechanism.	Reprinted from [30] with permission from MDPI
Potato protein	These proteins are categorized into three groups: patatins, which make up 40–60% and have molecular weight of 40–43 kDa; protease inhibitors, comprising 20–30% with molecular weights between 16 and 25 kDa; and a group of other proteins with high molecular weights.	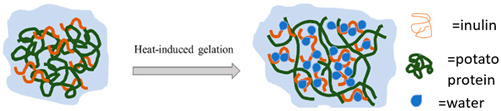 E.g., heat induced protein gelation in the presence of inulin.	Reprinted from [50] with permission from Elsevier
Rice protein	The protein composition includes glutelin (alkali-soluble, comprising 80% of the total, with molecular weight ranging from 60 to 600 kDa and subunits connected by disulfide bonds), globulin (salt-soluble, 12%, MW 12–20 kDa), albumin (water-soluble, 5%), and prolamin (alcohol-soluble, 3%).	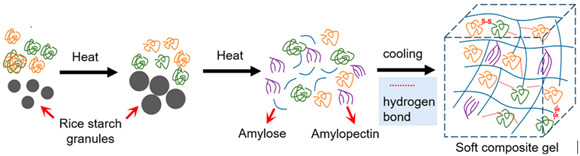 E.g., rice protein soft gel formation in the presence of rice starch.	Reprinted from [51] with permission from Elsevier
Soy proteins	Composed primarily of storage globulins, including glycinin (11S, hexamer, 320–380 kDa) and β-conglycinin (7S, trimer, 150–220 kDa). In glycinin, disulfide bonds link its basic and acidic subunits.	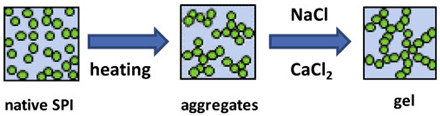 E.g., effect of heating, NaCl, and CaCl_2_ on aggregated gel formation.	[52] and reprinted from [53] with permission from Elsevier
Starch (cereal flour)	D-*α*, 1–4, 1–6-linked glucose polymer, mainly made of amylose (linear) and amylopectin (branched).	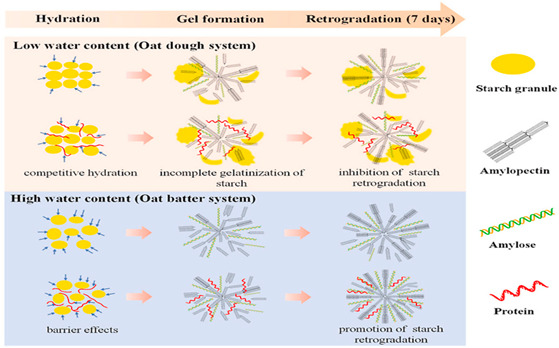 E.g., oat starch micro-crystallite gel formation at high and low water content.	Reprinted from [54] with permission from Elsevier
Xanthan gum	Polysaccharide chain consists of two *β*-D-glucose units linked through the 1, 4 positions. The side chain consists of two mannoses and one glucuronic acid, and thus the chain consists of repeating modules of five sugar units.	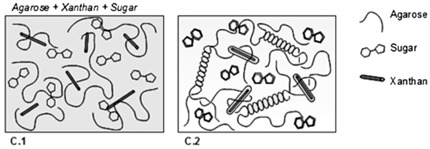 E.g., xanthan gum gel formation in the presence of sugar and agarose.	Reprinted from [55] with permission from Elsevier
Animal-based gels			
Bovine Serum Albumin (BSA)	The third most abundant whey protein in milk, comprising up to 10% of the total whey proteins. It contains 35 cysteine residues that form 17 disulfide bridges, with one free sulfhydryl group.	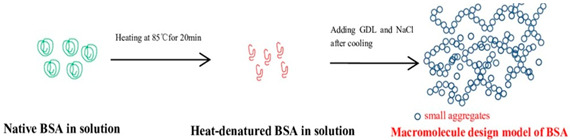	[56] and reprinted from [57] with permission from Elsevier
Casein	Casein is composed of four main types of proteins: αS1-casein, αS2-casein, β-casein, and κ-casein, which self-assemble into casein micelles. These micelles are stabilized by colloidal calcium phosphate and electrostatic interactions. A family of phosphoproteins make up about 80% of the protein content in cow’s milk.	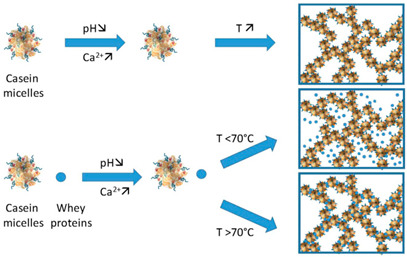 E.g., casein aggregation as induced by heat, calcium, pH, and whey protein.	[58] and reprinted from [59] with permission from Elsevier
Egg proteins	Consist of about 70% albumen (globular proteins containing ovomucin fibres) and 30% egg yolk (several types of low-density lipoproteins).	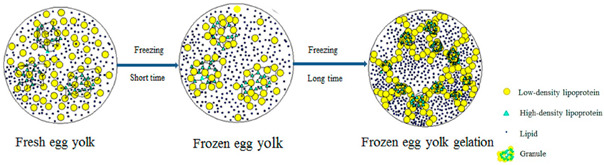	Reprinted from [60] with permission from Elsevier
Gelatin	Protein high in glycine and proline.	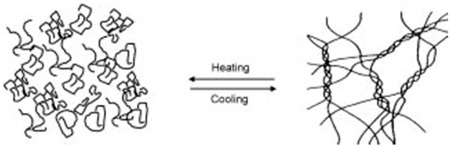 Sol-to-gel helix formation of protein as affected by temperature.	Reprinted from [61] with permission from Elsevier
Whey protein	Mainly comprise globular proteins like β-lactoglobulin and α-lactalbumin.	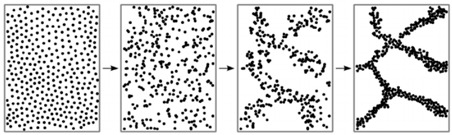 Aggregates of whey protein particle gels.	Reprinted from [28] with permission from Elsevier
Microbial-based gels			
Gellan gum	A pure culture of the microbe *Sphingomonas elodea* produces a polysaccharide with four connected simple sugars: one unit of rhamnose, one unit of glucuronic acid (a form of glucose that has been oxidized), and two units of glucose.	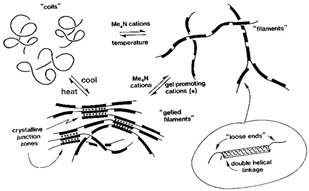	Reprinted from [62] with permission from Elsevier

When gellan gum is combined with high levels of co-solute, including sugars, glucose syrup, or polyols, the gelation mechanism shifts from enthalpic to entropic. As the co-solute concentration increases from 50 to 80% *w*/*w*, the molecular interactions are disrupted, resulting in distinct structural properties. Co-solutes compete for water molecules, reducing the availability of free water necessary for proper hydration and the formation of hydrogen bond-supported helices between gellan chains. As a result, the formation of extensive and stable helix-based junction zones is suppressed. This causes the gelation process to rely on lightly crosslinked gellan chains, where large parts of the molecule remain in the disordered conformation [62,63]. The macromolecular arrangement resembles that of a lightly vulcanized synthetic rubber, where the contribution to viscoelasticity is entropic via the dynamic reconfiguration of the chain segments between adjacent crosslinks (knots of the rubbery hydrocolloid structure). The outcome is a very different type of texture characterized by elasticity (reduced brittleness) and stretchability, similar to gelatin-made soft confections [63,64].

Other polysaccharides also form entropic networks of high elasticity under high-solid conditions. For example, κ-carrageenan has a molecular structure consisting of long chains of sulphated D-galactose and L-anhydrogalactose. A condensed system with κ-carrageenan at a concentration of 0.5–3% (*w*/*w*) was achieved when glucose syrup was used as a co-solute, with an overall solid content of 83% (*w*/*w*) [65] or polydextrose with a solid content of 85% (*w*/*w*) [25]. The addition of KCl at concentrations of 50–200 mM acts as a crosslinker in the system, resulting in the formation of lightly crosslinked strands according to the domain model [22]. In the case of high-methoxy pectin, which consists of binding blocks as a linear polymer of partly esterified α-(1–4)-linked D-galacturonic and mannuronic acids, gels are formed at a concentration of 3% (*w*/*w*) mixed with approximately 80% (*w*/*w*) glucose syrup at pH 3 (adjusted with 0.2 M HCl). The microstructure, observed under scanning electron microscopy, reveals a consistently smooth surface, while infrared spectroscopy indicates a structure similar to that of the co-solute, with no chemical bonds being formed between the gelling agent and co-solute [24,66,67].

In contrast, chitosan, a linear polysaccharide with randomly distributed β-(1→4)-linked D-glucosamine and N-acetyl-D-glucosamine, is used to prepare hydrogels by dissolving 3% (*w*/*w*) chitosan in 2% (*w*/*w*) acetic acid. The addition of trisodium phosphate (3.6–4.4% *w*/*w*) leads to the formation of covalent bonds between the phosphate groups and amine groups of the chitosan molecules, resulting in a non-crystalline structure with a high crosslink density. With an increasing concentration of TSP, the mesh size and molecular weight between crosslinks are reduced and eventually reach thermodynamic equilibrium, as described by the Flory–Rehner theory [34]. A similar occurrence was observed when the crosslinker was changed to genipin (2.5% *w*/*w*), which has carboxymethyl groups that bond with the amino groups of chitosan, resulting in a chemical structure change to a secondary amide. Wide-angle X-ray diffraction analysis reveals that the high-solid chitosan matrix has a non-crystalline structure. Genipin is distributed in the system, causing the unpacking of the polymeric chains, leading to the further repositioning of hydrogen bonds in the covalently crosslinked network [38].

Proteins can form gels through physical processes such as heat or pressure, which create a firm structure and texture. Naturally, heat helps to release exposed hydrophilic patches and embedded hydrophobic groups, resulting in an unfolded structure. In the case of high pressure at 600 MPa for 15 min at an ambient temperature, the partial denaturation of the protein solution occurred for soy glycinin, ovalbumin, and bovine serum albumin at concentrations ranging from 30 to 60% (*w*/*w*). When the concentration of these proteins was increased to 80% (*w*/*w*), the unfolding of the native conformation, primarily composed of β-sheets in the secondary structure of soy glycinin, was preserved [27,68]. In similar conditions, the high hydrophobicity and the bonds between the sulfhydryl and disulfide bonds of the ovalbumin molecule [27,69], the structure of the bovine serum albumin, which was a stable arrangement of disulfide linkages [27,69], and the secondary conformation of alpha helix or the beta sheet of immunoglobulins were also preserved [70].

The preservation of the structure of condensed whey protein 80% (*w*/*w*) can be achieved either by using high pressure at 600 MPa for 15 min at sn ambient temperature [71] or by evaporating water at temperatures not exceeding 40 °C from the system until the desired high concentration is reached [72]. Furthermore, the structure of the Amide I and Amide II bands was still present in the mixtures of 80% (*w*/*w*) whey protein with glucose syrup, and the hydrophobic binding sites were embedded in the original structure, which remained amorphous [73]. Studies have also shown that in another method of preservation, protein molecules retain their structure without any conformational changes provided that pressure or temperature exposure conditions of limited moisture content are met.

Gelatin is extracted from animal skins or bones, such as those of cattle or fish, using either acid (Type A) or alkali (Type B), which causes the breakdown of connective tissues in collagen. The amino acids that assist in gelation are proline and glycine [35]. Gelatin is useful in its ability to prepare high-solid slabs (80–85% *w*/*w*) through both physical and chemical preparations. In the first case, gelatin at a concentration of 15% (*w*/*w*) was dissolved in water then heated at a temperature not exceeding 50 °C. The solid content in the system was then increased by adding co-solute including glucose syrup or polydextrose. It was found that increasing the co-solute concentration to between 10 and 50% results in the formation of dense helicoidal strands of the protein, which give rise to an amorphous three-dimensional structure.

However, when the co-solute concentration was increased to 60–65% (*w*/*w*), phase separation occurred between the two components [74]. This phase separation became more evident when agarose (1.5% *w*/*w*) was added, revealing a phase behaviour characterized by non-interactive bicontinuous phases in the limited aqueous environment. Polydextrose restricted the formation of the gel network in agarose, while gelatin retained molecular ordering through the coil-to-helix transition in its gelled state [75]. In the case of crosslinking between gelatin and genipin (0.5–3% *w*/*w*), a high-solid film with a cohesive structure was obtained, resulting in covalent interactions due to the heterocyclic genipin-crosslinked gelatin and intermolecular hydrogen bonds. The gelatin network becomes denser and cohesive as the genipin concentration is increased [76,77,78].

Clearly, the above discussion demonstrates that condensed hydrocolloid formulations offer benefits that encompass improved texture, long-term stability in the shelf life of food products, as well as potential benefits like glycemic control, fat replacement, partial salt substitution, and advantages beyond normal nutrition. However, to optimize these deliverables in the overall product quality, the molecular constraints of hydrocolloid gels should be understood and overcome based on insights from the gelation theory for low-solid enthalpic networks, the free volume theory for high-solid entropic networks, and similarly, from the surface tension theory for emulsification and foamability. Taking advantage of molecular characteristics and the structural relaxation of hydrocolloids in the glassy state of condensed preparations would assist in preventing moisture loss and microbial growth, hence extending the shelf life of novel end products [70,79,80,81]. The successful incorporation of low-calorie insoluble and soluble fibre in high-solid materials without compromising organoleptic properties would help to control gastric emptying, fight Type 2 diabetes, and potentially lower plasma cholesterol levels.

## 3. Mechanical vs. Calorimetric Glass Transition Temperature in Condensed Food Gels

High-solid gels made of natural polymers and co-solute form largely amorphous matrices whose metastable state (at apparent equilibrium, despite being capable of changing to a thermodynamic stable state with crystalline consistency) determines their physicochemical, structural, and textural characteristics. This consistency is obtained when the temperature of the system is rapidly reduced, preventing its components from being organized into a crystalline lattice. The temperature at which a sample achieves glassy consistency is referred to as the glass transition temperature (*T_g_*) [80]. Unlike the well-defined melting point (*T_m_*), *T_g_* is less precisely defined because the vitrification process can occur over a broad temperature range [19,82,83]. This temperature is important in terms of technology and the biofunctional role of food systems. Specifically, at temperatures below *T_g_*, physical, chemical, and biological changes are limited due to the retardation in the movement of molecules [83]. When the temperature of the food system is above *T_g_*, several important physical changes occur. These include particle agglomeration, increased stickiness, crystal formation, and the collapse of microcapsule matrices that encapsulate internal particles. This rise in temperature also accelerates key chemical processes, such as Maillard reactions, lipid oxidation, and the degradation of vitamins. Additionally, it enhances enzymatic activity and promotes microbial growth, collectively influencing the stability and quality of the food product [84].

Measurement of the glass transition temperature can be accomplished using two primary methods: differential scanning calorimetry (DSC) and mechanical analysis (MA). The former tracks the heat flow related to structural transitions—such as changes between amorphous and crystalline states—in materials over time and temperature under controlled conditions. DSC provides both quantitative and qualitative insights into physical and chemical transformations, whether they involve heat absorption (endothermic) or release (exothermic), or shifts in the material’s heat capacity. The resulting thermograms show the difference in heat flow between the sample and an inert reference material as the temperature increases or time progresses [85]. For gels with a low water content, for instance 30% *w*/*w*, *T_g_* can be determined by observing the reduced mobility of the polymer’s main chain, followed by the gradual motion of its side chains and smaller molecular segments. This transition is typically reported as a temperature range encompassing three stages: the onset (*T_gi_*), midpoint (*T_gp_*), and endset (*T_ge_*) [18,86,87]. The accuracy of measurement is influenced by various factors, including the heating rate, sample size, conditioning, and moisture content [88,89,90].

The mechanical analysis, on the other hand, is used to track changes in metastable transitions, which describe high-solid gel systems with characteristics such as elasticity and viscosity, also known as viscoelasticity. Changes in the mechanical properties of the gel as a function of temperature are collected in the master or composite curve. These can be recorded by small deformation in shear, obtaining important rheological data that include the elastic component (*G*′)*,* the viscous component (*G*″), and tan *δ* (the ratio of *G*″ to *G*′). The parameters are used to classify the viscoelastic characteristics of the high-solid gel system into four phases: The glassy state (Phase I), where the value of *G*′ is very high, up to 10^10^ Pa, while that of *G*″ is lower. In this state, the gel is rigid because the molecular migration is very slow. The glass transition region (Phase II), where both modulus traces (*G*′ and *G*″) suddenly decrease to between 10^8^ and 10^5^ Pa. In the rubbery state, (Phase III), *G*′ is greater than *G*″, forming a soft gel with tan *δ* being less than 1.0. The flow region (Phase V), at relatively high temperatures, where the molecules begin to flow like a liquid and tan *δ* exceeds 1.0 [18,91].

The mechanical glass transition temperature is derived from the analysis of rhelogical properties, particularly through oscillatory shear measurements conducted in high-solid gels. During the controlled cooling of the sample, the three viscoelastic parameters of *G*′, *G*″, and tan *δ* are continuously monitored. The cooling profile typically exhibits an increase in both *G*′ and *G*″, with the maximum value of *G*′ signifying the transition of the material into the glassy state. Subsequently, the time–temperature superposition principle (TTS), a foundational concept in the study of glass-forming behaviour, is employed to further interpret the material’s rheological response [92,93]. It allows viscoelasticity predictions at very high/low temperatures and frequencies of oscillation, which are beyond the normal experimental constraints, thus showcasing the metastable nature of the glass with reduced frequency or time of observation [15].

By combining the *G*′ and *G*″ data from the entire cooling profile at a reference temperature (*T*), a parameter known as the shift factor (*α_T_*) is derived. This shift factor can be used to model data described by the reaction rate theory or the free volume theory. In the former, an Arrhenius law is obtained, which represents the thermodynamics of the statistical mechanics that compare the reaction rate constant (*k*) at an experimental temperature (*T*) with a reference condition (*k_o_*, *T_o_*), hence producing an activation energy (*E_a_*) for the molecular process (Equation (1)). The latter assumes the existence of free volume due to string-like vibrations of the polymer chains, hence yielding the concept of the fractional free volume (*f*) in Equation (2) (here, B is a constant equal to 1.0) [67]:

(1) $$\log\alpha_{T}=\frac{E_{a}}{2.303R}\left(\frac{1}{T}-\frac{1}{T_{o}}\right)$$

(2) $$log\alpha_{T}=\frac{B}{2.303}\left(\frac{1}{f}-\frac{1}{f_{o}}\right)$$

By assuming a linear relationship between the fractional free volume and thermal expansion coefficient (*a_f_*)

(3) $$f=f_{g}+\alpha_{f}(T-T_{o})$$

We can derive the Williams–Landel–Ferry (WLF) equation as follows [94]:

(4) $$log\alpha_{T}=-\frac{\left(B/2.303f_{0})(T-T_{o}\right)}{(f_{o}/\alpha_{f})+(T-T_{o})}$$

A graph of the shift factor on a log scale (*y*-axis) against temperature (*x*-axis) produces a discontinuity in the log *α_T_* pattern, which is labelled as the mechanical glass transition temperature (*T_gm_).*

This framework has been widely applied to estimate *T_gm_* across a variety of food matrices, as shown in Table 2. Modelling represents the state transition between the glassy state and the glass transition region in high-solid amorphous gels. Its value is influenced by multiple factors, including protein denaturation, moisture content, type and concentration of gelling agent, and the presence of co-solute. These components alter the mechanical *T_g_* by modifying the gel’s physical bonding structure through plasticization or anti-plasticization effects [95].

Plasticization occurs when small molecules, such as water or a lipid, mix into the gel system. Water affects the stability of the food, as shown by the Gordon–Taylor equation below, which demonstrates that adding water causes changes in the glass transition temperature:

(5) $$T_{g}=\frac{w_{1}\times T_{g1}+K\times w_{2}\times T_{g2}}{w_{1}+K\times w_{2}}$$

where *T_g_*_1_ is the glass transition temperature for food and T*_g_*_2_ for pure water, with the amount of water in the food system and pure water being *w*_1_ and *w*_2_, respectively. *K* is the specific constant of the equation for each food gel [96].

The addition of co-solute results in shielding the intra- and intermolecular hydrogen bonds and dipole–dipole interactions between polymer chains and water molecules. The insertion of essential fatty acids, such as oleic acid (omega-9), linoleic acid (omega-6), and linolenic acid (omega-3), affects the cohesion and strength of the polymeric network [65,67,97]. These fatty acids act as plasticizers, enhancing the mobility of the polymer chains, thus lowering both the mechanical and calorimetric *T_g_*.

**Table 2:** Mechanical and calorimetry *T_g_* values of high-solid food gels in the presence of bioactive compounds.

High-Solid Gel System	Bioactive Compound	Moisture Content	Calorimetry *T_g_* (°C)	Mechanical *T_g_* (°C)	*f*_g_	*E_a_* Matrix (KJ/mol)	Reference
2% high-methoxy pectin + 77.4% polydextrose	0.4% vitamin C	20.2%	−43.5	−20	0.040	203.00	[24]
3% high-methoxy pectin + 81% glucose syrup	1% oleic acid	15%	−37	−15	0.040	-	[67]
**2**% κ-carrageenan with 50 mM KCl + 82% glucose syrup	1% thiamin hydrochloride	15%	−35	−7	0.038	219.76	[98]
2% κ-carrageenan with 50 mM KCl + 82% polydextrose	1% α-linolenic acid	15%	−32	−8	0.042	**-**	[25]
1% κ-carrageenan with + 82% glucose syrup + 200 mM KCl	1.5% linoleic acid + 0.5% lecithin	15%	−28	−21	0.042	**-**	[65]
3% κ-carrageenan with + 80% glucose syrup + 200 mM KCl	1.5% linoleic acid + 0.5% lecithin	15%	−12	−2	0.042	**-**	[65]
2% κ-carrageenan with 50 mM KCl + 82% polydextrose	1% caffeine	15%	−32	0	0.042	318.2	[22]
2% κ-carrageenan with 200 mM KCl + 82% polydextrose	1% caffeine	15%	−20	10	0.041	333.7	[22]
80% whey protein isolate (atmospheric condition)	20% lactose	20%	-	−14	0.029	-	[71]
80% whey protein isolate (pressurized condition at 600 MPa, 15 min)	20% lactose	20%	-	−18	0.029	-	[71]
31.6% whey protein isolate + 47.4% glucose syrup (undenatured)	1% linoleic acid	20%	-	−35	0.040	-	[73]
79% whey protein isolate (undenatured)	1% linoleic acid	20%	-	−16	0.040	-	[73]
25% bovine gelatin + 59% glucose syrup	1% nicotinic acid	25%	−31	−14	0.042	269.1	[99]
25% fish gelatin + 59% glucose syrup	1% nicotinic acid	25%	−43	−34	0.042	226.0	[99]
20% bovine gelatin + 64% polydextrose + 0.25% genipin	0.75% linoleic acid + 0.25% lecithin	15%	−14	11	0.040	**-**	[26]
20% bovine gelatin + 64% polydextrose + 0.50% genipin	0.75% linoleic acid + 0.25% lecithin	15%	12	21	0.040	**-**	[26]

Anti-plasticization is a phenomenon where the glass transition temperature of high-solid gels increases due to changes in the composition. Ikasari et al. (2023) reported that increasing the amount of κ-carrageenan from 0.5% to 3% (*w*/*w*) in mixtures with glucose syrup, linoleic acid, linoleic acid, and potassium ions as co-solutes, at a total solid content of 85% (*w*/*w*), resulted in a shift in mechanical *T_g_* from −17 to −2 °C [65]. Additionally, increasing the concentration of potassium ions (50–200 mM KCl) in a similar system raised the mechanical *T_g_* from 0 to 10 °C. The phenomenon is due to the increased amounts of κ-carrageenan and potassium ion-induced electrostatic bonds in this system [65]. In the case of high-solid protein gels with glucose or polydextrose as co-solute (85% *w*/*w* total solids), it was found that bovine gelatin (Bloom 225) formed a stronger gel with a higher mechanical *T_g_* compared to fish gelatin (Bloom 65) [99]. Furthermore, reinforcing the bovine gelatin system by adding a natural crosslinking agent, genipin, strengthened the network and enhanced its stability in the presence of linoleic acid and lecithin, exhibiting a mechanical *T_g_* of 21 °C [29].

The prediction of the values of mechanical *T_g_* in relation to plasticization and anti-plasticization is a key fundamental in designing functional high-solid gels [38]. Such estimates could be extrapolated in vivo to reflect conditions in the gastrointestinal system and the diffusion process of bioactive compounds delivered from the gel [100]. Recent studies have revealed that mechanical *T_g_* shows a stronger correlation with the release behaviour of bioactive compounds and lipid oxidation compared to calorimetric *T_g_* [65,99] in model food systems.

The lower the volume fraction of the gel, the greater the extent to which the mechanical *T_g_* differs from the corresponding measurements of calorimetry. The latter appears to be insensitive to the macromolecular (network) morphology of the condensed hydrogel. Besides a thorough description of the material in terms of its composition or preparation history, the glass transition temperature depends on the analytical method and protocol employed. Thus, the discrepancies observed in the values of mechanical and DSC *T_g_* in Table 2 are not an experimental artefact but, rather, a reflection of the distinct property and distance scales being probed via the two techniques.

## 4. Utility of the Mechanical Glass Transition Temperature on the Techno- and Bio-Functionality of Food Gels

### 4.1. Effect on the Diffusion of Bioactive Compounds from Hydrocolloid Matrices

The stability of sensitive bioactive compounds within a high-solid gel is largely affected by the physicochemical transformation of the gel as a function of external stimuli, which determines the molecular migration of the bioactive compounds being encapsulated or entrapped in the system. Such a transformation also influences how the bioactive compounds interact with the gel matrix, potentially impacting their efficacy, shelf life, and overall functionality [88,90]. The structural tunability of various gel systems allows them to respond distinctly to environmental stimuli, such as pH, temperature, enzymatic activity, and ionic strength, facilitating the controlled release of micronutrients [101]. Multiple physicochemical and structural parameters govern the transport behaviour of bioactive compounds within biopolymer matrices, and a selection of these critical factors is summarized in Table 3 [15]. Of particular importance is the spatial migration of functional molecules, including vitamins, essential fatty acids, essential oils, antioxidants, and antimicrobials, within the gel matrix, which is essential for maintaining functionality and facilitating effective nutrient delivery [24,102,103,104].

**Table 3:** Multiple physicochemical and structural parameters that govern the transport behaviours of bioactive compounds.

Determining Factor	Properties	Reference
Polymer	Molecular arrangement (amorphous, crystalline, or semi-crystalline) Crosslinking between ionic molecules, cross-linker agents, and polymeric materials Glass transition (Decrease in *T*_g_ leads to higher diffusion rates due to increased segmental mobility of polymeric material) Macro- and microporosity Viscosity	[26,105,106]
Diffusant	Size of diffusing compound Crosslinking between penetrant and matrix	[106,107,108]
Plasticizer	Presence of water Low-molecular-weight solute	[15,109]
Temperature	Biopolymer relaxation (Increase in temperature leads to increase in free volume available for solute diffusion)	[22,110]
Geometry	Slab, cylinder, or sphere	[111,112]
Time	Diffusion duration	[24]
pH	Charge, electrotatic repulsion, and swelling behaviour of the matrix	[112,113]
Ionic strength	Ionization suppression and change in swelling behaviour	[114,115]

A study by Jiang & Kasapis (2011) followed the controlled release of caffein, a central nervous system stimulant, in model systems of 79.6% glucose syrup or 79.1% glucose syrup with 0.5% k-carrageenan (10 mM KCl) [23]. The role of the viscoelastic transformation of the gelling polysaccharide in controlling the diffusion of caffein near the glass transition temperature (*T_g_*) was clearly demonstrated. Similar results by Zhou & Roos (2012) indicated the stabilization of >95% and ~100% of thiamine hydrochloride (vitamin B1), in condensed lactose and trehalose systems, respectively, after 60 days of storage at −80 °C (below *T_g_* of matrices of −42 °C) [116]. The decrease in the available free volume significantly hindered the diffusion of bioactive compounds, due to the presence of freeze-concentrated solutes forming highly viscous glassy matrices that reduced the rates of segmental mobility and the chemical reaction of bioactive compounds at sub-*T_g_* temperatures.

Furthermore, extensive work has been carried out to elucidate the underlying mechanisms governing the release kinetics of a range of bioactive compounds from condensed food gels. These included vitamin B1 [98], vitamin B2 [117], nicotinic acid (vitamin B3) [99,118], vitamin B12 [119], ascorbic acids (vitamin C) [24], vitamin E [120], a-linolenic acid [25], oleic acid [67], linoleic acid [73], cinnamon essential oil [121], caffeic acid [23,122], and carvacrol [123] (Table 4). These studies reveal the capacity of the glass transition temperature of high-solid matrices in slowing down the diffusion of the above micronutrients. In ascorbic acid, for example, the extent of structural relaxation and molecular mobility within the glass transition region of a matrix made of 2% (*w*/*w*) high-methoxy pectin with 77.6% (*w*/*w*) polydextrose, played a significant role in defining the rate and extent of the vitamin’s molecular migration to release media.

In monolithic delivery systems where the bioactive compound is homogeneously distributed within the hydrocolloid gel, the release profile is predominantly governed by Fickian diffusion kinetics. This concept relies on the assumption that transport occurs without substantial alterations in the morphological properties of the matrix (e.g., constant nano-porosity, the absence of swelling or stationary boundaries, time-based permeability of the diffusant) [15,124]. Hence, for thin films with negligible edge effects, the diffusion equation is calculated as follows [124,125]:

(6) $$\frac{M_{t}}{M_{o}}=1-\frac{8}{\pi^{2}}\sum_{n=0}^{\infty }\frac{1}{{\left(2n+1\right)}^{2}}exp\left(\frac{-{D_{t}{\left(2n+1\right)}^{2}\pi }^{2}t}{L^{2}}\right)$$

with the early release approximation written as follows:

(7) $$\frac{M_{t}}{M_{o}}=4\sqrt{\frac{D_{t}}{\pi L^{2}}}for0\leq \frac{M_{t}}{M_{o}}\leq 0.6$$

and the late release approximation as follows:

(8) $$\frac{M_{t}}{M_{o}}=1-\frac{8}{\pi^{2}}\;exp\left(\frac{-\pi^{2}D_{t}}{L^{2}}\right)\;for0.4\leq \;\frac{M_{t}}{M_{o}}\;\leq 1.0$$

**Table 4:** Effect of glass transition on chemical and enzymatic activities.

Biopolymer Matrices	Active Compounds	Triggers for Gel Formation	Key Findings	References
A. Controlled delivery of bioactive compounds				
Saponin–chitosan	Vitamin A	Electrostatic interaction	Vitamin A trapped in a saponin–chitosan mixture was released quickly at pH 1.2, with up to 70% released within the first 30 min. The low pH caused the chitosan to become more positively charged, weakening its structure and facilitating the release of the vitamin, while also lowering the glass transition temperature from 77 °C to 65 °C.	[126]
High-methoxy pectin with polydextrose	Ascorbic acid (Vit C)	Hydrophilic interaction and presence of co-solute	Ascorbic acid diffused rapidly from a high-methoxy pectin and polydextrose matrix above its glass transition temperature, with mobility closely linked to free volume changes, and this behaviour was successfully modelled using a combination of Fickian diffusion and the modified Williams–Landel–Ferry (WLF) theory.	[24]
κ-carrageenan with glucose syrup	Thiamin hydrochloride (Vit B1)	Hydrophilic interaction and presence of co-solute	The controlled release of thiamin from a glassy κ-carrageenan/glucose syrup matrix was governed by non-Fickian diffusion and the modified Williams–Landel–Ferry (WLF) theory, highlighting the strong influence of the glass transition and polymer relaxation on vitamin mobility.	[98]
WPI microcapsules	Nicotinic acid (Vit B3)	Hydrophilic interaction and presence of co-solute	The diffusion of nicotinic acid in spray-dried whey protein microcapsules was governed by temperature-dependent transport modelled using combined Fickian diffusion and the modified Williams–Landel–Ferry (WLF) theory, with the mobility closely linked to the free volume and glass transition behaviour of the protein matrix.	[118]
Waxy maize starchmicrocapsules	Tocopheryl acetate (Vit E)	Heat treatment	The release of tocopheryl acetate from waxy maize starch microcapsules was influenced by temperature, with the diffusion mechanism being controlled by free volume changes within the matrix, and a relationship between the diffusion coefficient and fractional free volume was established using the modified WLF theory.	[120]
Bovine gelatin with glucose syrup and fish gelatin with glucose syrup	Nicotinic acid (Vit B3)	Hydrophilic interaction and presence of co-solute	Fish gelatin exhibited lower glass transition temperatures and more flexible matrix structures compared to bovine gelatin, which had higher *T_g_* values and a denser, more rigid network, resulting in the slower diffusion of nicotinic acid.	[99]
Dried starch/bentonite clay	Vitamin B2	Hydrophilic interaction	The *T_g_* of starch–clay composites shifted to higher temperatures with increasing vitamin B2.	[117]
Dried sodium alginate and poly(vinyl acetate)	Vitamin B12	Crosslinking (4% CaCl_2_ and glutaraldehyde) heat treatment	Vitamin B12 release was higher from alginate than from PVA scaffolds, with both systems exhibiting enhanced release rates at elevated temperatures due to increased activation energy.	[119]
k-carrageenan with polydextrose	A-linolenic acid	Presence of co-solute	Release mechanism of omega-3 fatty acid (α-linolenic acid) from a κ-carrageenan/polydextrose matrix demonstrated that the diffusion of omega-3 was governed by the free volume theory and significantly influenced by the glass transition temperature of the matrix, with less Fickian diffusion observed in the glassy state.	[25]
High-methoxy pectin with glucose syrup	Oleic acid	Presence of co-solute	Preservation of oleic acid within a high-methoxy pectin and glucose syrup matrix highlighted the impact of the glass transition temperature and structural relaxation on oleic acid diffusion and its stability in the matrix, with diffusion behaviour governed by the free volume and temperature-dependent processes.	[67]
Whey protein isolate with glucose syrup	Linoleic acid	Presence of co-solute	Diffusion of linoleic acid from whey protein matrices was affected by the glucose syrup concentration. The high concentration of glucose syrup reduced the glass transition temperature and increased the effective diffusion coefficient, enhancing the mobility of the fatty acid.	[73]
Condensed chitosan, starch, and pectin compounds	Rosemary (REO), mint essential oil (MEO), nisin, and lactic acid	Crosslinking and intermolecular interactions	REO, MEO, nisin, and lactic acid significantly increased flexibility, improved the water barrier properties (0.014 g.mm/m^2^ 24 h), tensile strength (25.95 MPa), and thermal stability, making them suitable for the controlled delivery of bioactive compounds in food packaging.	[104]
Polyvinyl alcohol—*Alyssum homolocarpum* seed gum	Nisin	Thermal gelation	The controlled release of nisin from PVA-AHSG composite films was modelled by a pseudo-Fickian diffusion mechanism. It highlighted the importance of the glass transition (*T_g_*) temperature for the thermal properties and controlled release, and showed that nisin diffusivity increases with concentration due to enhanced film hydrophilicity.	[127]
Gum arabic with inulin	Spicata essential oil (SEO)	Presence of co-solute	The microencapsulation of spearmint essential oil (SEO) using inulin and gum arabic revealed a Fickian diffusion-controlled release profile best modelled by the Peppas–Sahlin model, highlighting the importance of the glass transition temperature (*T_g_*) for understanding the matrix’s thermal properties and SEO release behaviour.	[128]
Sodium alginate, gelatin, gum acacia, and carboxymethylcellulose sodium	Cinnamon essential oil	Crosslinking CaCl_2_, electrostatic interaction, and co-solute (glycerol)	The release of cinnamon essential oils from calcium alginate films, followed Fickian behaviour, was influenced by the glycerol content, free volume, intermolecular interactions, solvent partition coefficient, and the glass transition temperature (*T_g_*).	[121]
K^+^-κ-carrageenan/polydextrose	Caffeine	Presence of co-solute	Caffeine diffusion from a K^+^-κ-carrageenan/polydextrose system was significantly influenced by the mechanical glass transition temperature (*T_gm_*), with an increased potassium ion concentration raising the *T_gm_*, which in turn decoupled caffeine diffusion from the structural relaxation of the matrix, leading to more efficient molecular transport.	[22]
Chitosan and collagen	Caffeic acid (CA)	Crosslinking (Tetraethyl orthosilicate)	The release behaviour of caffeic acid (CA)-loaded chitosan–collagen composite hydrogels was influenced by the glass transition temperature (*T_g_*), which increased from 60–70 °C to 70–90 °C in the presence of CA, hindering molecular chain movement and thus affecting the diffusion of CA through the hydrogel.	[122]
Soy protein isolate (SPI)	Carvacrol	Thermal gelation	The effective carvacrol diffusivities in SPI-coated papers was determined by the experimental release kinetics using Fick’s second law, increase in temperature, and relative humidity (RH). This increase is closely correlated with changes in the glass transition temperature (*T_g_*) of the protein matrix, which enhance molecular mobility in the rubbery state.	[123]
B. Lipid oxidation				
Gelatin with lactose	Methyl linoleate	Presence of co-solute	The oxidation of methyl linoleate encapsulated in an amorphous lactose–gelatin matrix highlighted how the glass transition temperature (*T_g_*) influences the rate of lipid oxidation, with higher oxidation rates occurring when the matrix undergoes crystallization at the elevated temperature, which releases the encapsulated oil.	[129]
Freeze-dried maltodextrin	Flaxseed oil	Thermal gelation	In freeze-dried maltodextrin matrices encapsulating flaxseed oil, the glass transition temperature (*T_g_*) did not show a direct relationship with the oxidation rate, but the physical collapse of the matrix, which occurred at high water activity (Aw), increased the exposure of the encapsulated oil to oxygen, thereby accelerating oxidation.	[130]
Gelatin with polydextrose	Linolenic acid	Presence of co-solute and crosslinked with genipin	The oxidation rate of linolenic acid in gelatin/polydextrose systems was significantly influenced by the mechanical glass transition temperature (*T_g_*), with higher *T_g_* values achieved through genipin crosslinking, leading to a reduction in the rate of lipid oxidation by limiting the molecular mobility.	[29]
κ-carrageenan/glucose syrup	Linoleic acid	Presence of co-solute and increased biopolymer concentration	In condensed κ-carrageenan/glucose syrup systems, the mechanical glass transition temperature (*T_gm_*) significantly influences the rate of lipid oxidation, with higher *T_gm_* values associated with reduced oxidation rates, as it restricts molecular mobility during the propagation phase of oxidation.The increase in κ-carrageenan resulted in an increase in the *T_gm_* of matrices.	[26,65]
C. Non-enzymatic browning (NEB)				
Freeze-dried lactose, trehalose, and lactose/trehalose	L-lysine and D- xylose	Thermal treatment	The rate of non-enzymatic browning (NEB) is influenced by the glass transition temperature (*T_g_*), with reactions occurring more slowly below the *T_g_* due to reduced molecular mobility, but accelerating above the *T_g_* as the molecular motion increases, particularly in systems where the crystallization of components like lactose and trehalose occurs.	[131]
Milk powder	Lysine	-	The rate of the Maillard reaction in milk powder is influenced by the glass transition temperature (*T_g_*), with the reaction rate decreasing near the *T_g_* due to limited molecular mobility, as higher viscosity in the glassy state reduces the mobility of reactants, thus slowing the reaction.	[132]
Dehydrated potato	Not specified	-	In dehydrated potato, the glass transition temperature (*T_g_*) and water activity (aw) significantly influence the rate of non-enzymatic browning (NEB), with the reaction rate increasing as the temperature surpasses the *T_g_* and the water activity rises. The crystallization of sugars and the presence of highly mobile water further enhance the NEB rate, especially at higher water activities.	[133]
Freeze-dried maltodextrin (MD) or polyvinylpyrrolidone (PVP)	L-lysine and D-xylose	Thermal treatment	In amorphous food models, the rate of non-enzymatic browning (NEB) is influenced by the glass transition temperature (*T_g_*), with higher NEB rates observed above the *T_g_*, particularly when the water activity increases, though the reaction can still occur at low temperatures in the glassy state, especially in systems with higher water content.	[134]
Amorphous maltose/whey proteinisolates	L-lysine and D-xylose	Thermal treatment	The rate of non-enzymatic browning (NEB) in an amorphous maltose/whey protein isolate matrix is influenced by the glass transition temperature (*T_g_*), with the reaction rate accelerating above the *T_g_* as the molecular mobility increases due to water sorption, while the presence of whey protein reduces NEB by lowering the molecular mobility.	[135]
D. Enzymatic activity				
Freeze-dried maltodextrin/sucrose and maltodextrin/lactose/sucrose	Invertase	Thermal treatment	Enzymatic activity, particularly sucrose hydrolysis via invertase, is affected by the glass transition, with the water activity playing a crucial role by enhancing reaction rates through increased molecular mobility from water.	[136]
Gellan/polydextrose/p-nitrophenyl-α-d-glucopyranoside (pNPG)	α-d-glucosidase	Presence of co-solute	As the temperature was reduced to the *T_g_* of matrices, the molecular mobility was significantly reduced, leading to a marked decrease in the activity of α-d-glucosidase.	[137]
Starch and maltodextrin	α-amylase	Presence of co-solute	The mechanical glass transition temperature (*T_g_*) significantly affects the enzymatic hydrolysis of starch and maltodextrin by influencing the mobility of the enzyme reactant, with activity being significantly reduced below the *T_g_* due to the decreased molecular mobility.	[20]
Spherical freeze-dried whey protein isolate (WPI)	α-glucosidase	Thermal treatment	The mechanical *T_g_*, which greatly impacts the enzymatic activity, was notably diminished below the *T_g_*.	[138]

In the case of a spherical matrix with diffusion in the radial direction, the corresponding equation is as follows:

(9) $$\frac{M_{t}}{M_{o}}=1-\frac{6}{\pi^{2}}\sum_{n=1}^{\infty }\frac{1}{n^{2}}exp\left(\frac{-{D_{t}^{2}\pi }^{2}t}{R^{2}}\right)$$

Here, *M_t_* and *M_o_* represent the cumulative amounts of the bioactive compound released at time *t* and at 0, respectively, *n* is a fitting parameter, *D_t_* denotes the diffusion coefficient at time *t*, *L* refers to the film thickness, and *R* is the diameter of the sphere.

To delve deeper into the mechanistic interpretation of the molecular migration in high-solid systems, one needs to consider the so called ‘hole free volume’ within a polymer network [139]. The concept of free volume, initially introduced for amorphous synthetic polymers, underscores the critical role of intermolecular voids in facilitating the string-like vibrations of polymer chains [140]. For diffusion to occur, the voids must be of sufficient size to accommodate the displacement or ‘jumping’ of molecules into adjacent sites. In highly crystalline matrices, the molecular mobility is severely constrained due to the tightly packed and regularly ordered arrangements, whereas in amorphous systems, the degree of crosslinking serves as the dominant factor regulating diffusion [141,142].

Diffusing compounds must acquire sufficient thermal energy to overcome attractive forces, suggesting that the molecular ‘jump’ mechanism is primarily governed by thermal processes and the associated activation energy. Molecules confined within the lattice-like structure of polymeric chains rely on activation events that facilitate energy transfer throughout the system. When the supplied energy surpasses a critical threshold, molecules can undergo displacement, initiating a sequence of random walks and successive movements that are influenced by the availability of free sites within the polymer matrix. In contrast, if the energy is insufficient, molecules remain immobilized in their initial position [143,144].

In the case of vitamin C, progression in release kinetics was calculated with the reaction rate theory of Equation (1), with an activation energy of 7.6 kJ/mol in comparison to the *E_a_* of a 2% (*w*/*w*) high-methoxy pectin with a 77.6% (*w*/*w*) polydextrose matrix of around 200 kJ/mol [24]. For concentrated 79% (*w*/*w*) whey protein isolate with glucose syrup, *E_a_* was 206 kJ/mol, i.e., much higher than the omega-6 linoleic acid (*E_a_* = 42 kJ/mol). Therefore, the vitrification of the polymer and its associated energy of activation is distinct from the diffusivity of the bioactive compound, hence serving as a crucial indicator of the system’s physical state and its ability to modulate bioactive compound release. Above the glass transition temperature, the segmental mobility of the polymer progressively facilitates the diffusion of small molecules, highlighting the critical role of structural relaxation in this process.

The mathematical expression that describes the progress in the free volume in relation to diffusion kinetic is as follows [145]:

(10) $$-loga_{T}=log\left[\frac{D\left(T\right)}{D\left(T_{g}\right)}\right]=\frac{C_{1g}^{\prime }(T-T_{g})}{C_{2g}^{\prime }+(T-T_{g})}$$

For *T* > *T_g_*,

(11) $$C_{1g}^{\prime }=\xi C_{1g}\;and\;C_{2g}^{\prime }=C_{2g}$$

By combining Equations (3), (7), (10) and (11), a mathematical expression can be established between the diffusion coefficient above the glass transition temperature and the corresponding changes in the free volume [139]:

(12) $$\log D(T)=\log D(T_{g})+\frac{\xi }{2.303}\left[\frac{1}{f_{g}}-\frac{1}{f}\right]$$

where *f* and *f_g_* are the fractional free volume at *T* and *T_g_*, respectively, and *ξ* is the coupling parameter that represents the relationship between the critical molar volume of the jumping unit of a bioactive compound and that of the polymer matrix [146]. A linear correlation was observed when plotting log[*D*(*T*)] against (1/*f_g_* − 1/*f*), from which the coupling parameter (*ξ*) was derived. This parameter, therefore, indicates the relationship between the structural relaxation of the polymer and the mobility of the diffusant.

**Figure 2:**
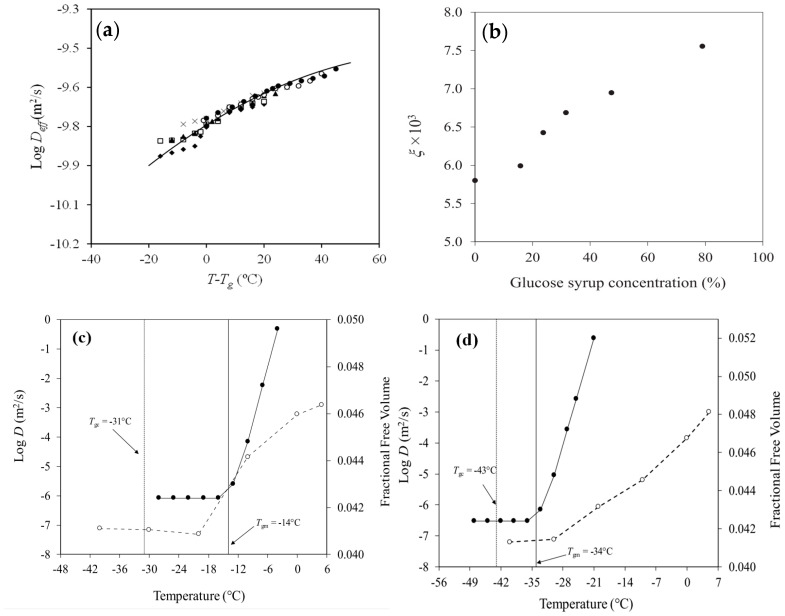
(**a**) Diffusion coefficient (*D*) of 1% linoleic acid in 79% solid matrix comprising whey protein isolate with percent substitution of the protein with glucose syrup 0% (◆), 20% (☐), 30% (▲), 40% (×), 60% (**○**), and 100% (●) as a function of *T* − *T_g_*, (**b**) the coupling parameters, *ξ*, of 1% linoleic acid release from 79% whey protein/glucose syrup matrices as a function of co-solute concentration (%), and the fractional free volume (●, right *y*-axis) and diffusion coefficient (*D*) of 1% nicotinic acid (**○**, left *y*-axis) from matrices of (**c**) 25% bovine gelatin with 59% glucose syrup and (**d**) 25% fish gelatin with 59% glucose syrup. Reprinted from [72,99,146] with permission from Elsevier.

The progression of the coupling parameter was followed by the diffusion of linoleic acid in a matrix with 79% total solids, comprising whey protein isolate that was substituted with glucose syrup from 0% to 100% [73]. The findings revealed a polynomial increase in the release rate of the fatty acid with temperatures rising above the glass transition temperature (*T* − *T_g_*), corresponding to the incremental substitution with glucose syrup (Figure 2a). The matrix exhibited a progressive decrease in activation energy from 335 to 193 kJ/mol, attributed to the plasticizing effect of both glucose syrup and linoleic acid [73]. This reduction in *E_a_* due to a decrease in the critical molar volume of the matrix was reflected as an increase in the value of the coupling parameter between the polymer and diffusant in the malleable mixture (Figure 2b) [72].

The molecular transport of vitamin B6 (2% *w*/*w*) within high-solid hydrocolloid gels reinforced with genipin, a natural crosslinking agent, was examined to manipulate their structural and functional properties. These were gelatin/co-solute (92% *w*/*w* total solids), bovine serum albumin/co-solute (63% *w*/*w* total solids), and whey protein isolate/co-solute (63% *w*/*w* total solids). Increasing the genipin concentration (0.5 to 3% *w*/*w*) resulted in a progressive reduction in the effective diffusion coefficient of vitamin B6, registering, for example, a value of 6 × 10^−11^ m^2^/s for BSA. This reduction in molecular transport is attributed to the substantial decrease in the network mesh size, which was reduced, for example, from 70 to 4 nm for WPI [77,147,148]. Findings confirm that genipin-mediated crosslinking effectively modulates the microstructural architecture of protein gels, thereby enabling precise control of the diffusion kinetics of encapsulated/entrapped bioactive compounds in high-solid foods.

Furthermore, in selecting the most suitable analytical approach to characterize the transport behaviour of bioactive compounds within food matrices, a comparative assessment of calorimetrically and rheologically determined glass transition temperatures was undertaken. The apparent diffusion coefficient was compared to the fractional free volume for mixtures of 1% nicotinic acid in 25% bovine gelatin with 59% glucose syrup and 1% nicotinic acid in 25% fish gelatin with 59% glucose syrup in the vicinity of the glass transition temperature. The type and molecular weight of the protein determined the vitrification pattens in these systems, with the mechanical and DSC glass transition temperatures of bovine gelatin (bloom 225, Mw = 173 kDa) being higher than those of fish gelatin (bloom 85, Mw = 60 kDa) (Figure 2c,d).

A significant increase, by approximately five orders of magnitude, was observed in the diffusion of nicotinic acid as the experimental temperature traversed the mechanical glass transition temperature of −14 °C, i.e., the diffusion rate increased from 4.84 × 10^−8^ m^2^/s at −20 °C to 1.26 × 10^−3^ m^2^/s at −5 °C in the bovine gelatin matrix (Figure 2c). Similarly, a pronounced enhancement in the values of the diffusion coefficient for the bioactive compound was monitored around the mechanical glass transition temperature of −34 °C, i.e., the diffusion rate increased from 7.60 × 10^−8^ m^2^/s at −40 °C to 9.90 × 10^−4^ m^2^/s at −21 °C of the fish gelatin matrix in Figure 2d [99]. Hence, it is the network connectivity (*T_gm_*) rather than the change in the heat capacity of solids (*T_gc_*) that play a dominant role in regulating the molecular dynamics involved in the diffusion of nicotinic acid.

Overall, the mechanical or network glass transition temperatures provide useful insights into the molecular diffusion of bioactive compounds in high-solid hydrocolloid gels for the rational design of controlled release systems. The composition of the supporting matrix has a pronounced influence on its mechanical rigidity, restriction in free volume, and diffusional behaviour. Delivery vehicles formulated with WPI, BSA, gelatin, κ-carrageenan, or high-methoxy pectin in the presence of sugars and polyols such as sucrose, glucose syrup or polydextrose, and crosslinking agents like KCl or genipin exhibit diverse structural configurations governed by the nature of molecular interactions and the total solid content. These compositional variations significantly affect the encapsulation or entrapment efficiency and the kinetics of nutrient release, with matrices exhibiting higher crosslinking density or tighter molecular packing in the vicinity of *T_gm_*, demonstrating the superior quality control of diffusion processes that can now be rationalized on a fundamental basis.

### 4.2. Effect on the Rate of Lipid Oxidation in High-Solid Food Systems

The previously discussed extensive research has underscored the critical role of the glass transition temperature in modulating the molecular mobility of bioactive compounds, which also exerts a significant influence on the kinetics of chemical reactions in food products, particularly lipid oxidation (Table 4). The degradation of lipids primarily occurs via two distinct mechanisms: hydrolytic and oxidative rancidity. The former entails the enzymatic or moisture-induced hydrolysis of ester bonds in triacylglycerols and phospholipids, resulting in the formation of free fatty acids, monoacylglycerols, diacylglycerols, and lysophospholipids. This process is typically catalyzed by lipolytic enzymes or facilitated by the presence of water under specific storage conditions [149]. Oxidative rancidity, commonly referred to as lipid autoxidation, is initiated by unsaturated fatty acids reacting with molecular oxygen via a free radical-mediated, autocatalytic chain reaction [150].

In low-moisture systems, oxidative reactions are frequently diffusion-limited due to the restricted mobility of oxygen and other reactive species. Lipid oxidation can be substantially attenuated by encapsulating unsaturated lipids within oxygen-impermeable amorphous matrices. When such systems are stored below their glass transition temperature, the constrained molecular mobility and reduced permeability of oxygen or pro-oxidants markedly inhibit the progression of oxidative degradation [151].

This process involves the formation of lipid hydroperoxides (LOOH), which are the initial products of lipid peroxidation, and function as key intermediates in the breakdown of unsaturated lipids. It proceeds through a well-established triphasic mechanism encompassing initiation, propagation, and termination stages, each contributing to the progressive deterioration of the quality and safety of lipid-rich food matrices [152]. During the initiation phase, alkyl radicals (L•) are generated via hydrogen atom abstraction from (poly)unsaturated fatty acids (LH), a process typically catalyzed by external stimuli such as thermal energy, ultraviolet radiation, or transition metal ions [153]. In the initiation phase, also referred to as the induction period (IP), the concentration of lipid hydroperoxides increases in a nearly linear manner following a pseudo-zero order reaction [154].

In the propagation phase, these alkyl radicals rapidly react with molecular oxygen to form peroxyl radicals (LOO•), which subsequently abstract hydrogen atoms from additional LH molecules, yielding new LOOH and L• species [155]. Given the minimal depletion of LH in bulk lipid systems, the kinetics of LOOH formation can be approximated by a pseudo-first-order model. At elevated oxygen levels, the steady-state concentration of L• becomes negligible, and LOO• radicals predominate, leading to termination predominantly via LOO• self-recombination reactions [156]. Subsequently, the accumulated LOOH species undergo decomposition through a bimolecular branching mechanism governed by pseudo-second-order kinetics. The overall accumulation of LOOH reflects the dynamic interplay between its formation and decomposition. The application of integrated kinetic models, in conjunction with the linear initiation profile, enables the extraction of kinetic parameters, offering an informative assessment of oxidative stability compared to conventional single-parameter models [157].

Modelling follows the initiation phase of LOOH by estimating the rate constant for a zero-order reaction (*k*_1_, meq kg^−1^ h^−1^) as follows [158]:

(13) $$\frac{d[LOOH]}{dt}=k_{1}$$

The rate constants for the pseudo-first-order (*k_f_*, meq kg^−1^ h^−1^) and pseudo-second-order (*k_d_*, meq kg^−1^ h^−1^) reactions, corresponding to the formation and decomposition of lipid hydroperoxides during the propagation phase, are also determined. The kinetic data are modelled using a sigmoidal fitting approach corresponding to the propagation phase of LOOH accumulation [156].

(14) $$[LOOH]=\frac{k_{f}}{\exp\left[k_{f}\left(a-t\right)\right]+k_{d}}$$

where *a* is an integration constant (mM^−1^) and *t* is time (h). From this equation, the rate of formation (*k_f_*) and decomposition (*k_d_*) of lipid hydroperoxides is obtained and further plotted against the mechanical and calorimetric glass transition temperatures.

**Figure 3:**
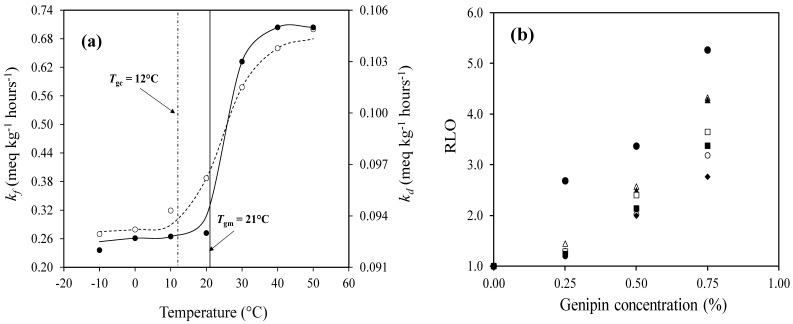

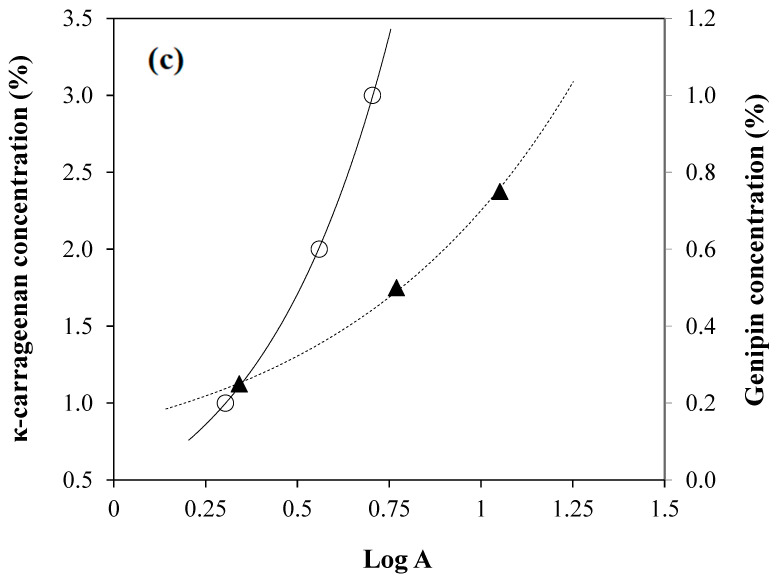
(**a**) Rate constant of ROOH formation (*k_f_*) (**○**, left *y*axis) and decomposition (*k_d_*) (●, right *y*-axis) in the propagation phase of 20% bovine gelatin/64% polydextrose/0.75% linolenic acid/0.25% lecithin/0.5% genipin, with reference to both mechanical (*T_gm_*) and calorimetric (*T_gc_*) glass transition temperatures; (**b**) the relative inhibition of lipid oxidation (RLO) in bovine gelatin/polydextrose/linolenic acid/lecithin matrices as a function of genipin concentration was recorded at various temperatures: −10 (●), 0 (△), 10 (▲), 20 (☐), 30 (■), 40 (**○**), and 50 °C (◆); and (**c**) oxidation resistance (A) measured for different κ-carrageenan concentrations in a glucose syrup/linoleic acid/lecithin system (**○**, left *y*-axis) and for varying genipin concentrations in a bovine gelatin/polydextrose/linolenic acid/lecithin system (▲, right *y*-axis). Reprinted from [26,29] with permission from Elsevier.

A recent study of linolenic acid (omega-3 fatty acid) oxidation within a system comprising 20% bovine gelatin, 64% polydextrose, and 0.25% lecithin demonstrated the temperature dependence of the rate constants for hydroperoxide formation and decomposition. The findings emphasize the importance of the mechanical glass transition temperature over the calorimetric glass transition temperature in controlling the lipid oxidation rates (Figure 3a) [29]. The *k_f_* consistently exceeded the *k_d_*, with *k_f_* decreasing from 0.65 to 0.32 meq kg⁻^1^ h^−1^ and *k_d_* decreasing from 0.104 to 0.094 meq kg^−1^ h^−1^ near the *T_gm_*, while at the lower *T_gc_* predictions, both parameters remained constant at their lowest value. The lipid hydroperoxide accumulation rate significantly diminished in the glassy state, highlighting the dominant role of the *T_gm_* in regulating oxidative kinetics in condensed gels.

It was further demonstrated that the lipid oxidation rates were strongly influenced by the physicochemical nature of the material. Specifically, in the presence of genipin from 0.25 to 0.75% (*w*/*w*), the formation of a dense network through genipin-crosslinked gelatin significantly delayed the onset of oxidation, as evidenced by the prolonged initiation/induction periods (*IP*) calculated using Equation (15) [158]:

(15) $$IP=\frac{k_{f}\left(2-k_{f}a+\ln k_{d}\right)-4{\left[LOOH\right]}_{0}k_{d}}{4k_{1}k_{d}-{k_{f}}^{2}}$$

where [*LOOH*]_0_ denotes the initial concentration of hydroperoxides at time zero.

This structural reinforcement near the mechanical glass transition temperature restricts the molecular mobility and limits oxygen diffusion, thereby suppressing oxidative reactions [26]. The findings underscore the critical role of genipin–gelatin crosslinking in mitigating lipid oxidation, with higher degrees of crosslinking correlating with improved oxidative stability in glassy matrices. This is reflected in the increased relative inhibition of lipid oxidation (*RLO*) calculated using Equation (16), and plotted in Figure 3b as a function of genipin concentration [159]:

(16) $$RLO=\frac{{IP}_{n}}{{IP}_{o}}$$

where *IP_o_* and *IP_n_* represent the induction/initiation periods in the absence and presence of crosslinkers (e.g., genipin) at various concentrations.

To further demonstrate the oxidation resistance of the hydrocolloid matrix (*A*), Equations (17) and (18) were employed. Here, *R_or_* is the ratio of the oxidation rate calculated from the initiation constants of peroxidation in the absence (*k*_10_) and presence (*k*_1*n*_) of various crosslinkers in the food gel:

(17) $$R_{or}=\frac{k_{1n}}{k_{10}}$$

(18) $$A=\frac{RLO}{R_{or}}$$

Figure 3c demonstrates that increasing the genipin concentration markedly improved the oxidative resistance (*A*) of linolenic acid within gelatin/polydextrose/lecithin matrices containing 85% (*w*/*w*) total solids [26]. This enhancement is consistent with the observed increase in the relative inhibition of lipid oxidation (*RLO*), which can be attributed to the formation of an extensively crosslinked genipin–gelatin network [29] reducing molecular mobility and limiting oxygen diffusion, ultimately stabilizing the essential fatty acids that are crucial for various bodily functions.

Looking further into low-moisture foods, including crackers (made, for example, with all-purpose flour, salt, and baking soda), studies on lipid (i.e., soybean oil) oxidation in relation to the surface and internal lipids indicate that the former are no more susceptible to oxidation than the internal counterparts, suggesting uniform oxygen exposure throughout the end product. This phenomenon is due to the highly porous structure of crackers, which facilitates oxygen diffusion throughout the material [160]. In contrast, when gelatin (5% *w*/*w*) was introduced into a 70% (*w*/*w*) lactose to investigate the oxidation of methyl linoleate in amorphous food models (mimicking that of milk powder or coffee whiteners), the findings revealed that the methyl linoleate, while encapsulated by gelatin within the amorphous matrix, was effectively protected from oxidation [129].

A parallel effect was noted in the κ-carrageenan/glucose syrup systems (total solid 85% *w*/*w*) containing linoleic acid (omega-6 fatty acid), where the oxidative resistance (*A*) improved with increasing concentrations of potassium counterions (1–3% *w*/*w*). This outcome is attributed to enhanced ionic crosslinking and electrostatic interactions between κ-carrageenan chains and K^+^, leading to a more compact polysaccharide network, according to the domain model, which similarly restricts oxidative degradation [26,65]. This type of study provides evidence that advances the understanding of the relationship between the structural characteristics of condensed hydrocolloid gels and the kinetics of lipid oxidation for innovative functional food applications.

However, once the gelatin/lactose system underwent crystallization, particularly above its glass transition temperature at approximately 50 °C, the encapsulated oil was released and subsequently rapidly oxidized. The glass transition temperature proved to be a critical parameter, as amorphous materials become less stable and more permeable above this threshold. The work further demonstrated that time, moisture, and temperature significantly influence crystallization and lipid oxidation, with higher values of these external stimuli accelerating both mechanisms. It was concluded that lipid oxidation in dried foods is closely tied to physical changes in the amorphous matrix, particularly lactose crystallization, highlighting the importance of considering *T_g_* in the design of accelerated shelf life tests and strategies for improving product stability [129].

Overall, the literature underscores the importance of controlling the diffusion dynamics and maintaining storage conditions below the glass transition temperature to enhance the oxidative and physicochemical stability of low-moisture foods. The interplay between the properties of the glassy matrix and environmental factors is therefore central to designing food products with extended shelf life and improved resistance to quality degradation. Even in systems with potentially higher oxygen diffusivity, the inhibitory effect on oxidation may be enhanced in a matrix of glassy consistency, as determined by the mechanical *T_g_*. Factors such as porosity, phase separation, matrix collapse, and the degree of amorphous–crystalline interplay influence oxygen accessibility to lipid substrates or the mobility of the lipid itself. These are aspects that can be manipulated with an understanding of the utility of the glass transition temperature in high-solid food gels [129,161].

### 4.3. Effect on Non-Enzymatic Browning (NEB) in Condensed Food Systems

Non-enzymatic browning constitutes a major class of chemical reactions in condensed food systems, the kinetics of which are intricately influenced by the phenomenon of glass transition (Table 4) [80]. This chemical process is primarily promoted by thermal treatments and encompasses a broad range of settings, including the Maillard reaction, caramelization, the chemical oxidation of phenolic compounds, and maderization [162]. Among these, the Maillard reaction is characterized as a complex series of heat-induced events between reducing sugars and free amino acids or peptides, leading predominantly to the formation of melanoidins. It facilitates the browning of food at lower temperatures compared to caramelization, with its rate influenced by the pH, heat treatment, and water activity, reaching a maximum at intermediate *a_w_* values of approximately 0.6–0.7. The browning reaction proceeds more rapidly under alkaline conditions than in acidic environments and is strongly dependent on both time and temperature [163]. In addition, this non-enzymatic reaction is directly regulated by the glass transition, which is associated with alterations in food structure and is predominantly governed by diffusion-controlled processes [131]. Given that glass transition is highly dependent on water content and can occur under ambient conditions at a specific water activity, its role in controlling NEB is closely linked to the plasticizing and solvent function of water in high-solid systems [80,164].

A study conducted by Miao and Roos (2005) systematically examined the influence of the glass transition temperature on the kinetics of non-enzymatic browning in low-moisture food preparations composed of lactose, trehalose, and lactose/trehalose binary matrices, each incorporating L-lysine and D-xylose at a concentration of 5% (*w*/*w*) as reactants [131]. The extent of NEB was quantified by measuring optical density (OD), and the rate constant (*k*) was quantified using zero-order kinetics (*A* = *k_t_* + *A*_0_). Across all three systems, the glass transition temperature ranged from 26.6 °C to 28.8 °C at 33.2% RVP and from 9.6 °C to 11.9 °C at 44.1% RVP. The NEB rate observed at 44.1% RVP was markedly higher than at 33.2% RVP, highlighting the significant effect of environmental moisture on reaction kinetics. Increasing the RVP levels enhanced both water sorption and non-enzymatic browning, with the trehalose (a non-reducing sugar)/reactant system exhibiting NEB rates approximately twice as much as those of the other matrices [131].

Microstructural properties further influenced the reaction dynamics, as the freeze-dried trehalose/reactant system exhibited enhanced porosity and increased surface area, facilitating greater molecular mobility and reactant accessibility [165]. At elevated RVP (54.5% and 65.6%), the crystallization of component sugars, particularly lactose and trehalose, was initiated at the beginning of the rubber-to-glass transition upon cooling and was accompanied by a concentration of the amorphous phase, which in turn accelerated NEB. The rapid progression of NEB in the trehalose/reactant matrix (4.68 ± 0.08 OD units/g solid/h) was attributable to its superior water sorption prior to crystallization and propensity to form dihydrate crystals that preferentially excluded and concentrated reactants within the amorphous phase. Therefore, crystallization served as a critical driver for enhancing NEB by increasing the local component concentration and molecular reactivity within the remaining amorphous matrix. RVP exerted a direct control over NEB progression by modulating the depression of the glass transition temperature and promoting crystallization phenomena, thus underscoring the critical interplay between transitions in the physical state and the chemical reactivity of low-moisture food systems [131].

A study by Acevedo et al. (2008) explored the influence of glass transition and water–solid interactions on the kinetics of non-enzymatic browning in dehydrated potatoes. It was observed that below the *T_g_* in the glassy state, browning was still detectable [133]. Thus, NEB could occur in the glassy state in conjunction with low water activities of *a_w_* = 0.11 and 0.22, but the browning rates significantly increased in the supercooled state with enhanced molecular mobility [133]. The glass transition temperature decreased with increasing water content, and the difference between storage temperature (*T*) and the *T_g_* was a critical parameter in explaining non-enzymatic browning. When *T* − *T_g_* increased, particularly above 40 °C (*a_w_* = 0.84), the NEB rate reached its maximum around 0.65 ΔL*/h compared to 0.10 ΔL*/h at *T* − *T_g_* = −15 °C; ΔL* represents the luminosity of the colour developed from the NEB reaction.

Overall, the literature argues that above the *T_g_*, the mobility of hydrocolloids and co-solutes in the gel significantly accelerates the browning reactions due to increasingly mobile water fractions, detected through NMR relaxation times and DSC, an outcome that indicates a transition from bound to free water. Therefore, the integrated analysis of *T_g_* depression, water sorption behaviour, and molecular mobility confirm that the glass transition acts as a key limiting factor for NEB kinetics in low-moisture systems. This is not, however, an absolute barrier, especially when local heterogeneities and residual mobility are present in the condensed matrix [133,166].

Karmas, Buera, and Karel (1992) modelled the influence of glass transition on the rates of non-enzymatic browning in dehydrated vegetables and model systems composed of amino acids and sugars using the Williams, Landel, and Ferry equation in the following form [167]:

(19) $$log\frac{k}{k_{o}}=-\frac{C_{1}^{0}(T-T_{0})}{C_{2}^{0}+(T-T_{0})}$$

where *C*_1_^0^ and *C*_2_^0^ are the WLF constants evaluated at the reference temperature, *T*_0,_ and *k* and *k*_0_ correspond to the browning rate constants at the experimental and reference temperatures.

Below the *T_g_*, non-enzymatic browning proceeded slowly due to restricted molecular mobility, as indicated by low absorbance increases at specific wavelengths. For example, in lactose/CMC/xylose/lysine systems, absorbance at 280 nm proceeded extremely slowly below the of *T_g_* (~50 °C) at 0.1–0.2 OD units/g solid/h, confirming diffusion-limited conditions. Once the system temperature exceeded the *T_g_* by about 5 °C, a marked increase in browning was observed of about 0.7 OD units/g solid/h. This result supported the WLF position of sharply decreased viscosity and increased molecular mobility near the *T_g_*.

An Arrhenius equation was employed next, as follows [168]:

(20) $$k=Ae^{-(E_{a}/RT)}$$

where *E_a_* represents the activation energy necessary for a system to transition between conformational arrangements within the glassy state, while *R* denotes the universal gas constant (8.314 J mol^−1^ K^−1^). In doing so, the activation energy was estimated using plots showing a clear discontinuity corresponding to distinct kinetic zones. Below the glass transition temperature, the reaction kinetics were low but increased above the *T_g_* reflecting the enhanced sensitivity to temperature due to free volume expansion, and at temperatures well above the *T_g_*, the typical browning kinetics resumed. The results strongly validated the relevance of the WLF theory to NEB in amorphous food matrices. The rapid increase in reaction rates within a narrow temperature range above the *T_g_* highlighted the critical role of molecular mobility, as described by the free volume theory in conjunction with the glass transition temperature.

Collectively, these findings highlight the importance of considering the thermophysical conditions of low-moisture hydrocolloid gels and food systems, particularly their state transitions around the *T_g_* when modelling data, in order to predict or mitigate the chemical degradation caused by non-enzymatic browning. Integrating glass transition parameters into predictive models can substantially improve the accuracy of shelf life and quality deterioration forecasts in response to temperature fluctuations, and this is something that requires further consideration and development in the field.

### 4.4. Effect on Enzymatic Activity in Condensed Food Systems

Enzymatic reactions within food matrices are a critical factor contributing to physicochemical degradation and product deterioration, with their progression governed by a range of parameters that influence both the structural integrity and catalytic function of the enzyme [169]. Empirical studies involving invertase [136] and tyrosinase [170] have elucidated the sensitivity of enzymatic stability to variations in moisture content and the glass transition temperature.

For invertase (Kouassi & Roos, 2000), elevated water activity (*a_w_*) within the range of 0 to 0.54 and increasing moisture content from 23.9% to 76.4% have been demonstrated to significantly compromise sucrose stability within a sucrose–lactose matrix, indicating enhanced susceptibility to hydrolytic degradation under high humidity conditions [136]. The rate of sucrose hydrolysis increased with *a_w_* as the moisture content increased and the system transitioned from the glassy to the rubbery state, diminishing the diffusion barriers and leading to pronounced acceleration in the reaction rates. Water thus plays a dual role, i.e., facilitating structural organization and enhancing the diffusivity of both the enzyme and substrate within the matrix.

The experimental data in the above systems indicate that the reaction rate does not significantly change at the point where the temperature equals the glass transition temperature (*T* − *T_g_* = 0 at approximately *a_w_* 0.333). However, a notable increase in enzymatic activity is observed beyond *a_w_* 0.538, coinciding with the onset of crystallization. This suggests that crystallization may further enhance the molecular mobility and substrate diffusibility within the amorphous water–solute phase. The transition from a diffusion-limited glassy matrix to a more mobile rubbery or partially crystalline state facilitates enzyme–substrate interactions, thereby accelerating reactions such as sucrose hydrolysis [136,169].

Similar results were obtained for tyrosinase activity in low- and high-molecular-weight polyvinylpyrrolidone (PVP-LMW and PVP-K30, respectively), showing a stability profile closely aligned with the system’s glass transition temperature. Beyond *T* − *T_g_* = 0 °C, the rate constants for tyrosinase activity increase rapidly from 0.5 to 1.3 d^−1^, suggesting that *T_g_*-dependent mobility plays a central role in enzyme degradation kinetics [170]. Further elevation in the thermal conditions promotes structural destabilization, which consequently impairs enzymatic activity.

To further study the enzymatic activity vs. glass transition temperature in a complex carbohydrate matrix, an investigation was carried out using gellan/polydextrose mixtures loaded with α-d-glucosidase and p-nitrophenyl-α-d-glucopyranoside (pNPG) [137]. The enzymatic activity was observed to follow a clear temperature-dependent pattern. As the temperature was reduced to the *T_g_* of the matrices, the molecular mobility significantly decreased, leading to a marked decrease in the activity of α-d-glucosidase being monitored by its ability to hydrolyse the substrate, pNPG. The enzyme activity at temperatures below *T_g_* remained stable at the lowest value. Thus, the enzymatic activity in the gellan/polydextrose matrix significantly dropped from 0.639 nanokatals/g at above the *T_g_* to 0.344 nanokatals/g at −8 °C and −14 °C, both below the *T_g_*, confirming its efficient control of the enzymatic activity in glassy matrices.

In a further study of starch and maltodextrin matrices, the activity of α-amylase was observed [20]. The enzymatic activity was measured via the rate of glucose release, and it showed a marked reduction when the system entered the glassy state. The decrease in enzymatic activity correlated with the molecular rigidity imposed by the glass transition, where the diffusional mobility of both the enzyme and substrate is significantly hindered. At temperatures below the *T_gm_* of starch (−8 °C), the enzymatic activity dropped sharply from 7 to 0.5 μM glucose/s/g wheat starch. Similar results were also observed for a 77.5% *w*/*w* maltodextrin matrix (*T_gm_* = −22 °C), with the enzymatic activity dropping from 3 to about 0 μM glucose/s/g sample, suggesting that the enzyme’s ability to interact with the substrate was drastically reduced upon transitioning to the glassy state.

The influence of this mechanical parameter on the enzymatic activity was further scrutinized with the predictions of the reaction rate theory in the temperature range surrounding the *T_g_*. This approach articulates the intricate relationship between the temperature and parameters such as the steady shear viscosity (μ), reaction rate (*k*), or microbial inactivation during thermal treatment, as follows [20,171]:

(21) $$\log\left(\frac{k_{o}}{k}\right)=\frac{E_{a}}{2.303R}\left(\frac{1}{T}-\frac{1}{T_{o}}\right)$$

where *k* represents the reaction rate at a given temperature *T* (in Kelvin), *k*_o_ is the rate constant at a reference temperature, *T_o_*, *E*_a_ denotes the activation energy (expressed in J/mol), and *R* is the gas constant (8.314 J/mol K). By plotting log (*ko/k*) against 1/*T*, the model yields a straight line, where the slope (*E_a_/R*) allows for the determination of the activation energy. The plots revealed a discontinuity in the linear fits, suggesting the occurrence of the glass transition temperature. *E*_a_ calculations revealed that below the *T_g_*, starch exhibited a much higher activation energy (8.1 kJ/mol) compared to above the *T_g_* (0.8 kJ/mol), indicating a slow enzymatic reaction due to the lack of molecular mobility in the glassy state [20].

There is also literature on an enzymatic activity study of α-glucosidase in a spherical freeze-dried whey protein isolate (WPI) [138]. The study highlights the critical influence of the *T_g_* on the kinetics of enzymatic reactions in carbohydrate and protein matrices. The molecular dynamics of biopolymer networks, influenced by their respective *T_g_*, play a vital role in modulating enzymatic activity. At temperatures below the *T_g_*, the reduced molecular mobility results in hindered enzyme–substrate interactions, leading to lower reaction rates and enzyme efficiency. The findings emphasize the importance of considering the *T_g_* in the design of carbohydrate/protein-based matrices, especially where enzyme activity must be optimized for product stability and efficacy in low-moisture, amorphous foods and pharmaceuticals (Table 4).

Glassy transition modelling provides a mechanistic explanation, wherein decreasing the water availability induces structural rigidity in the enzyme environment, thereby diminishing its catalytic efficiency. This effect is largely due to the essential roles of water in facilitating substrate solubilization, preserving the enzyme’s native conformation through adequate hydration and enabling the molecular motions required for effective enzyme–substrate interactions. Furthermore, reduced hydrogen bonding in low-moisture environments leads to diminished enzyme solvation, adversely impacting structural stability and functional performance [172]. Under such conditions, the kinetics of diffusion-limited enzymatic reactions generally exhibit Arrhenius-type temperature dependence. Assuming that the enzyme maintains its bioactive conformation, these reactions can be effectively modelled using diffusion-based frameworks, suggesting the potential for the prolonged control of enzymatic stability in shelf-stable food products.

## 5. Conclusions

This review deals with the fundamental basis of addressing specific food applications following the incorporation of bioactive ingredients in condensed biomaterials made of natural hydrocolloids and co-solute. In doing so, it identifies the effect of vitrification and, in particular, the concept of the mechanical glass transition temperature as a critical determinant of bio- and techno-functionality in these jellified mixtures. This is a rapidly evolving field, but it appears that the concept of free volume combined with experimental observations on the diffusion rates of bioactive compounds, elimination of organic hydroperoxide formation, and minimization of the appearance of enzymatic/non-enzymatic end products can assist in the development of delivery vehicles or functional materials with improved nutritional value. The mechanical glass transition temperature can serve as a guide for better understanding the end product performance via molecular migration kinetics or carrier–diffusant interactions during processing and prolonged shelf life. Its informed manipulation with physical treatment or enzymatic/covalent crosslinking can enhance the bioavailability of stimulants to the central nervous system or retard the undesirable appearance of malodours byproducts, thus meeting consumers’ demand for wellbeing alongside improved sensory attributes. To further expand the application of the mechanical glass transition temperature (*T_g_*) on food gels in order to enhance their functional properties, future strategies should focus on the precise control of polymer composition, crosslinking density, moisture content, and the incorporation of plasticizers or nanoparticles in the delivery vehicle or protecting matrix. Suitable rheological tools, including basic rheometers and texture profile analysers (TPAs) found in the industrial setting, should be employed to correlate the shifts in the values of the mechanical glass transition temperature with the rigidity of the glassy state, thus enabling targeted gel design aligned with consumer expectations for prolonged techno- and bio-functionality. Such a fundamental outlook on future directions can inform the design of functional foods and nutraceuticals with improved likelihood of acceptance by the consumer.

## Data Availability

No new data were created or analyzed in this study.
